# Effects of seasonality and environmental gradients on *Spartina alterniflora* allometry and primary production

**DOI:** 10.1002/ece3.3494

**Published:** 2017-10-16

**Authors:** Troy D. Hill, Brian J. Roberts

**Affiliations:** ^1^ Louisiana Universities Marine Consortium Chauvin LA USA

**Keywords:** aboveground production, allometry, belowground production, nutrients, salinity, salt marsh

## Abstract

Predictions of how salt marsh primary production and carbon storage will respond to environmental change can be improved through detailed datasets documenting responses to real‐world environmental variation. To address a shortage of detailed studies of natural variation, we examined drivers of *Spartina alterniflora* stem allometry and productivity in seven marshes across three regions in southern Louisiana. Live‐stem allometry varied spatially and seasonally, generally with short stems weighing more (and tall stems weighing less) in the summer and fall, differences that persist even after correcting for flowering. Strong predictive relationships exist between allometry parameters representing emergent stem mass and mass accumulation rates, suggesting that *S. alterniflora* populations navigate a trade‐off between larger mass at emergence and faster rates of biomass accumulation. Aboveground production and belowground production were calculated using five and four approaches, respectively. End‐of‐season aboveground biomass was a poor proxy for increment‐based production measures. Aboveground production (Smalley) ranged from 390 to 3,350 g m^−2 ^year^−1^ across all marshes and years. Belowground production (max–min) was on average three times higher than aboveground; total production ranged from 1,400 to 8,500 g m^−2 ^year^−1^. Above‐ and belowground production were both positively correlated with dissolved nutrient concentrations and negatively correlated to salinity. Synthesis: Interannual variation in water quality is sufficient to drive above‐ and belowground productivity. The positive relationship between nutrients and belowground production indicates that inputs of nutrients and freshwater may increase salt marsh carbon storage and ecosystem resilience to sea level rise.

## 1 INTRODUCTION

Salt marshes are highly productive ecosystems (Chmura, Anisfeld, Cahoon, & Lynch, 2003). Their disproportionately high rates of carbon fixation make them valuable in attenuating atmospheric CO_2_, and their productivity and complex structure provide the foundation for estuarine food webs (Boesch & Turner, 1984; Odum & de la Cruz, 1967; Peterson, Howarth, & Garritt, 1985). The centrality of primary production to dynamics in coastal ecosystems has propelled efforts to understand the magnitude of salt marsh primary production and the forces driving it, and to predict how productivity will respond to environmental change.

Salt marsh net aboveground primary production (NAPP) responds to a variety of environmental conditions. Over a broad spatial scale spanning the Gulf and Atlantic coasts of North America, NAPP generally increases with temperature and growing‐season length (Kirwan, Guntenspergen, & Morris, 2009; Turner, 1976) and increases with tidal flooding until a physiological stress threshold is reached (Morris, Sundareshwar, Nietch, Kjerfve, & Cahoon, 2002). NAPP is also mediated by local environmental factors, such as nutrient availability (Valiela, Teal, & Sass, 1975), salinity (Snedden, Cretini, & Patton, 2015), and herbivory (Schultz, Anisfeld, & Hill, 2016; Silliman & Zieman, 2001). Predicting how environmental change affects salt marshes requires understanding the relative importance of productivity drivers, an effort aided by manipulative experiments and natural‐gradient studies but largely without verification from longitudinal data that can capture effects of ambient environmental variation at fixed locations.

Allometric relationships between stem height and mass are an important tool for nondestructively measuring biomass, enabling the collection of long‐term data while minimally impacting the system (Morris & Haskin, 1990). Changes in mass–height allometry produced by environmental gradients and other phenomena may affect the accuracy of this tool, although there is uncertainty about whether and why allometric relationships may shift.

Estimates of the magnitude of salt marsh primary production and its response to environmental change are limited both by mechanistic uncertainties and a shortage of high‐resolution data on marsh response to real‐world variation. To remedy these gaps, we gathered 3 years of allometry and biomass data from seven *Spartina alterniflora* Loisel marshes in coastal Louisiana, where salt marshes are exceptionally productive (Kirwan et al., 2009). Our objectives were to (1) explore temporal and spatial variation in *S. alterniflora* allometry, (2) report trends in biomass and evaluate insights from multiple production estimation methods, and (3) establish relationships between productivity and environmental conditions.

## 2 MATERIALS AND METHODS

### 2.1 Research marshes

**Figure 1:**
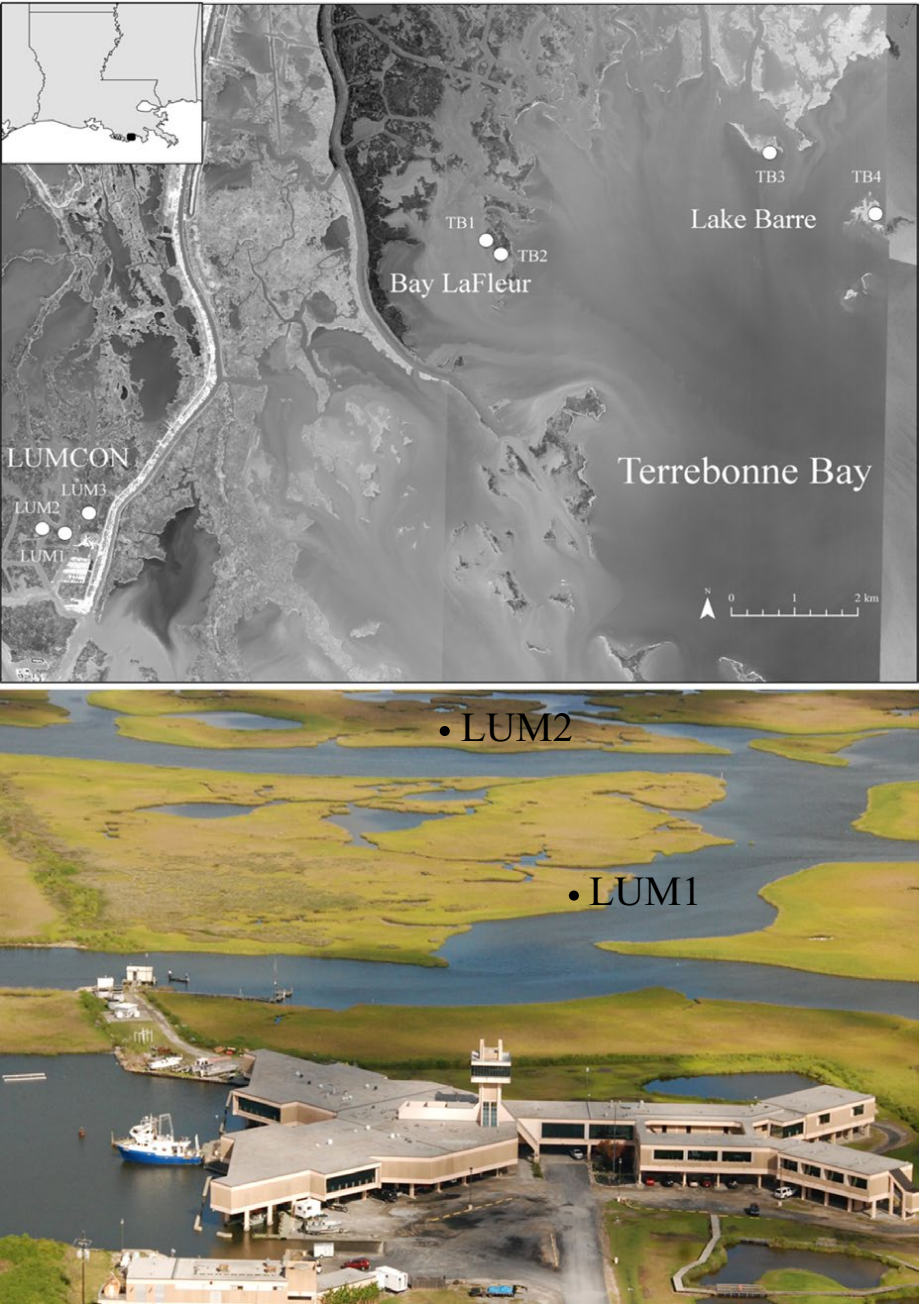
Top panel: Map of research marshes in three regions (LUMCON: LUM1, LUM2, LUM3; Bay La Fleur TB1, TB2; Lake Barre: TB3, TB4) near Terrebonne Bay, LA. Imagery provided by ESRI basemap. Bottom panel: Aerial photograph of salt marshes in the LUMCON region (research laboratory in foreground); LUM3 is out of view to right (north) in photograph

Seven salt marshes dominated by *S. alterniflora* spread among three regions of Terrebonne Bay, Louisiana (Figure 1), were chosen for this study. The LUMCON region is located in Cocodrie, LA region, near the DeFelice Marine Center of the Louisiana Universities Marine Consortium and is characterized by extensive areas of salt marsh, whereas marshes in Bay La Fleur and Lake Barre are on islands surrounded by open water. Soil characteristics of Bay La Fleur and Lake Barre salt marshes have been previously documented (Marton & Roberts, 2014; Marton, Roberts, Bernhard, & Giblin, 2015), and the Lake Barre region in particular has been a hot spot of vegetation loss for the past several decades (DeLaune, Nyman, & Patrick, 1994; Nyman, DeLaune, Roberts, & Patrick, 1993).

Two marshes in each region were sampled beginning in 2013, with an additional LUMCON marsh added in 2014. At each marsh, sampling occurred in three plots near permanent markers installed 20 m from the seaward marsh edge, in areas dominated by *S. alterniflora*. The experiment has a nested structure—three plots in each marsh, and two or three marshes per region. Sampling occurred monthly at the three LUMCON marshes. At Bay La Fleur and Lake Barre, sampling occurred monthly during 2013 and approximately quarterly thereafter. The middle of the month was targeted for all sampling events. Other species observed at these marshes include *Juncus roemarianus*,* Distichlis spicata*,* Spartina patens, and Avicennia germinans,* none of which were present in our monospecific plots.

Coastal marshes in Louisiana, like most of the Gulf Coast, experience low tidal amplitude (e.g., ~0.3 m; NOAA station 8762928, Cocodrie, LA), with wind and larger‐scale meteorological forcing often drive inundation (Turner, 2001). Water temperatures in the LUMCON region (station located 0.1–0.5 km from marshes; http://weatherstations.lumcon.edu/index.html) ranged between 5 and 35°C (mean: 22°C), and Lake Barre (near TB4) ranged between 7 and 32°C (mean: 23°C; CRMS 0355; https://www.lacoast.gov/crms_viewer2/Default.aspx#) from 2013 to 2015. Salinity ranged between with 2–22 (mean: 11) for LUMCON and 9–27 (mean: 18) for Lake Barre during the same period.

### 2.2 Allometry and biomass

At each sampling event, three 25 × 25 cm plots were haphazardly placed within a meter of the three permanent plot markers on the marsh platform, sampling over an approximately ten square meter sampling zone parallel to the seaward marsh edge. One quadrat was destructively harvested and two were measured nondestructively. Stems in the destructively harvested quadrat were cut at the sediment surface and processed in the laboratory. Live and dead stems were separated based on the presence of photosynthetic tissue. Stems were rinsed free of sediment and epiphytes, the total height of the plant was measured, and individual stems were dried to constant weight at 70°C. Beginning in May 2015, stems and leaves were weighed separately, and inflorescences and infructescences, when present, were measured and weighed separately.

Stems in two additional plots were measured nondestructively in the field, and surface litter was collected from all plots, if present. Litter was typically present in small quantities (see Results), and efforts were made to avoid repeated sampling of the same areas, minimizing but not eliminating the potential for litter collection to alter the microenvironment of future sampling plots. Litter was rinsed of salt and sediment and dried to 70°C.

Cores were also collected from the destructively harvested plot for analysis of belowground biomass (6.9 cm dia. × 30 cm). The 30‐cm‐depth interval was selected based on literature from the region (Darby & Turner, 2008b) and deemed appropriate by an assessment of root depth distributions. Belowground biomass was measured by rinsing peat free of sediment and drying to constant weight at 70°C. Beginning in May 2015, live and dead biomass were separated following Darby and Turner (2008b). Live belowground biomass during the period preceding direct measurement was estimated based on the strong relationship between live and total biomass (*r* ^2* *^= .71, *n* = 40; Fig. S1).

### 2.3 Water and soil properties

At each sampling event, salinity and water temperature were measured in the channel or bay adjacent to each marsh using a YSI sonde, and a water sample was collected for nutrient analysis. Water samples were filtered through acid‐cleaned (10% HCl) 0.2‐μm pore‐size 47‐mm‐diameter filters and stored frozen until analysis for dissolved inorganic nutrients (NO_3_^‐^+NO_2_^‐^, NH_4_^+^, PO_4_^3‐^, SiO_2_) using a Lachat Instruments QuickChem^®^ FIA+ 8000 Series Automated Ion Analyzer with an ASX‐400 Series XYZ Autosampler using standard techniques (Roberts & Doty, 2015).

Surficial sediment cores (6.9 cm dia. × 5 cm depth) were collected from the destructively harvested plot for measurement of bulk density, organic matter, organic C, total N and P. Surface cores were weighed wet, then subsampled to determine moisture content by drying at 80°C and organic content by mass loss on ignition. Bulk density was calculated as the dry mass of the core divided by the core volume. Beginning in November 2014, sediment temperatures were measured using a NIST calibration‐traceable partial immersion thermometer that displayed an integrated measure of sediment temperatures over the top 5 cm.

Remaining soil was dried, grounded with mortar and pestle, and passed through 2‐mm‐mesh sieve. Subsamples were placed in a glass desiccator and fumigated with concentrated HCl vapors for 24 hr to remove inorganic C. Samples were then analyzed for total organic C and total N using a CE Elantech Flash 1112 Elemental Analyzer. We ran concurrent sediment standards (National Institute of Standards and Technology [NIST], Buffalo River Sediment, 2704), which yielded organic C recoveries of 100.2 ± 0.4%. Total P was extracted by combusting soils (~0.2 g) with 0.1 ml of a 50% (w/v) solution of Mg(NO_3_)_2_ at 550°C for 1.5 hr and then shaking for 16 hr with 10% HCl. Supernatant was analyzed for PO_4_‐P concentrations as described above. Sediment standards for total P (NIST, Estuarine Sediment, 1646a) were digested and analyzed concurrently with samples and yielded a mean recovery of 99.4 ± 0.8%.

### 2.4 Allometry and productivity estimation

Measured stem heights (*h*; cm) were regressed against measured stem masses (*m*; g) using power relationships of the form *m *= *a*·*h* ^*b*^, where *a* and *b* are constants. The coefficient “*a*” describes the mass at unit height (1 cm), and the scaling exponent “*b*” describes how rapidly mass proportionally increases per unit height.

To determine whether regions and/or seasons (spring: March–May; summer: June–August; fall: September–November; winter: December–February) had distinct allometry, data were pooled into models for live or dead stems. Region‐ and season‐specific residuals were compared using ANOVA, approximating a nonlinear ANCOVA procedure (Snedden et al., 2015). Where region or season main effects were significant, subgroups were modeled separately.

Variation in mass–height allometry was evaluated on a continental scale using a similar approach. A pooled dataset was built using allometry data collected in Louisiana (present study), South Carolina (Morris & Haskin, 1990), Maryland (Lu et al., 2016), and Rhode Island (T. Hill, unpublished; C. Wigand, unpublished). Data were reduced to samples gathered during June–August for this comparison. State‐specific residuals from the pooled model were tested for significance using ANOVA.

The contribution of flowering to variation in stem allometry was examined using 2015 data. Allometry was compared among datasets (1) using total plant heights and masses (not distinguishing flower masses and heights), (2) excluding flowering plants entirely, and (3) correcting flowering plants for the height and mass of flowers/seeds.

Final allometry equations were applied to estimate biomass in the plots that were not destructively harvested. Stem masses were summed for each plot to calculate standing crops of live and dead biomass, averaged over the three plots per marsh. Net aerial primary production (NAPP) was calculated from marsh averages using five approaches:

1. peak live standing crop, where peak biomass is treated as total annual production;
2. end‐of‐season live (EOSL) standing crop (September biomass; e.g., Visser, Sasser, & Cade, 2006);
3. Milner and Hughes (1968), which sums positive live biomass increments and equals peak standing crop if senescence is complete and a single biomass maximum occurs;
4. Smalley (1958), which calculates production from incremental positive changes in live and dead biomass;
5. Valiela et al. (1975), which emphasizes the use of dead biomass increments to calculate NAPP under assumptions of balance between plant growth and mortality;

Net belowground primary production was calculated using the Milner‐Hughes, Smalley, and Valiela methods, and as the difference between maximum and minimum live biomass.

Differences between years and production estimation methods were quantified using two‐way ANOVAs run separately for each region. Where main effects were significant (*p* < .05), Tukey HSD post hoc test identified significantly different groups.

### 2.5 Identification of productivity drivers

To assess potential productivity drivers, we applied principal component analysis (PCA) to ancillary data on soil and water quality parameters, averaged over the main growing‐season period (April–October) for each year. Variables were scaled to unit variance prior to PCA to avoid bias from disparate parameter units. The two synthetic PCA dimensions were used in generalized linear models (GLM) with Smalley NAPP and max–min NBPP as response variables and the PCA dimensions as predictors. All analyses were performed in R ver. 3.3.3.

## 3 RESULTS

### 3.1 *Spartina alterniflora* allometry

Live stem densities tended to be slightly higher at LUMCON marshes than in the other regions (mean: 370 live stems per m^2^ vs. 296–323), although variation in stem densities was substantial and differences were not significant (one‐way ANOVA). Maximum stem heights reached 1–1.4 m and tended to be higher in the LUMCON region than elsewhere, although median stem heights in all regions were within 3 cm (48–51 cm). Median stem masses were also similar between regions (medians: 1.3–1.4 g/stem).

Despite general similarities in stem heights and masses, there were differences in mass–height relationships. Region and season were significant sources of variation in allometry of live stems (season: *F*_3,3644_ = 56.3, *p *<* *.001; region: *F*_2,3644* *_= 5.4, *p *<* *.01; Table S1), and a significant interaction term indicated that seasonal shifts in live‐stem allometry varied between regions.

The final allometry models used to estimate live‐stem masses were specific to each region (*n* = 3) and season (*n* = 4; Table S2) and had *r* ^2^ values between .32 and .81. Region‐ and season‐specific live‐stem models yielded residuals 22% lower than a single pooled model. Allometry of dead stems showed neither seasonal nor regional variation (Table S3), so dead‐stem masses were estimated from a single pooled model.

In 2015, the year when flowering was monitored, 64 harvested plants flowered (~2.5% of total). Flowering began in September and continued through the fall. Flowers increased stem height more than mass and therefore affected allometry by reducing the exponential term (Fig. S2). The effect of flowering could be corrected for by subtracting the mass and length of the inflorescence or infructescence from the mass and height of the plant, yielding allometry comparable to a dataset without any flowering plants. However, flowering did not affect the seasonality in allometry described above; seasonal differences persist whether flowering stems are included, excluded, or corrected (Tables S4 and S5). All subsequent calculations presented here are based on an unmodified allometry dataset, including flowering stems.

**Figure 2:**
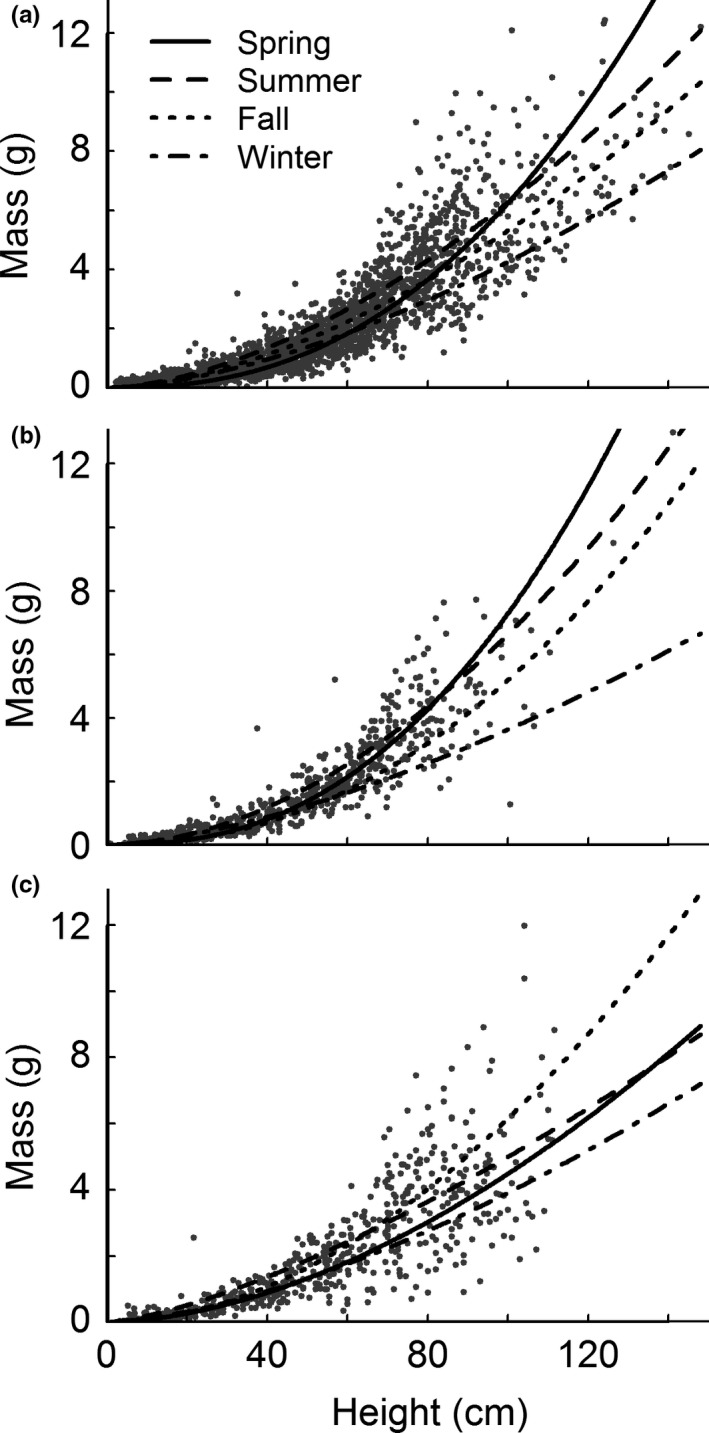
Seasonality in live‐stem allometry at LUMCON (panel a; *n* = 4013), Bay La Fleur (b; *n* = 1,008), and Lake Barre (c; *n* = 926). Lines show models for individual seasons (see Table S2 for regression parameters)

The practical effect of differences in allometry is that in each region, live stems shorter than 70–90 cm tended to weigh more in summer and fall than in winter and spring (Figure 2). In the LUMCON and Bay La Fleur regions, tall (>90 cm) stems had highest mass in spring, and masses of tall stems declined over the course of the year. This is in contrast to Lake Barre, where the pattern was more complicated; tall stems weighed most in fall and least in winter.

**Figure 3:**
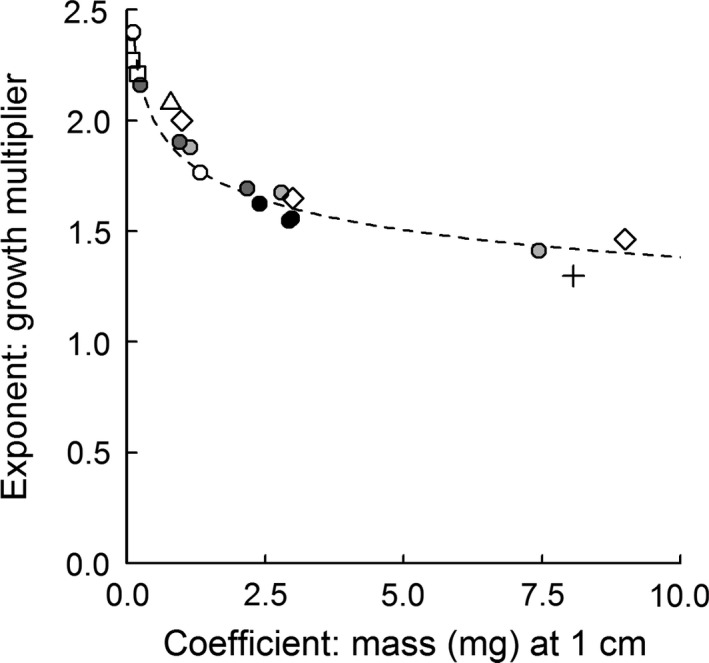
Relationship between allometry exponents and coefficients (see Table S2). Filled circles are data from present study; progressively darker colors correspond to seasons, spring–winter. Two spring data points overlap at *x* = 0.0001, *y* = 2.4. Open squares are from Thursby et al. (2002); open diamonds are from Hatcher and Mann (1975); open triangle is from Trilla et al. (2013); addition symbol is from Hopkinson, Gosselink, and Parrondo (1980). Line of best fit based on allometry from present study only: *y* = 0.79·*x* ^−0.12^, *r* ^2* *^= .98, *p *<* *.001

Across all regions, the allometry coefficient *a* was lowest in spring, and lower in fall than in winter and summer, although the fall decline at LUMCON was minimal. Stems emerging in summer and winter were between 2 and 29 times heavier than in the spring. Allometry exponents were all greater than 1.4, indicating increasing marginal mass gains per unit height in each season and region. In each region, higher exponents in spring and fall indicate greater mass gains per unit height during those seasons. Our allometry equations suggest a negative exponential relationship exists between allometry coefficients and exponents, reflecting a trade‐off between mass at unit height and the rate of mass accumulation (Figure 3).

**Figure 4:**
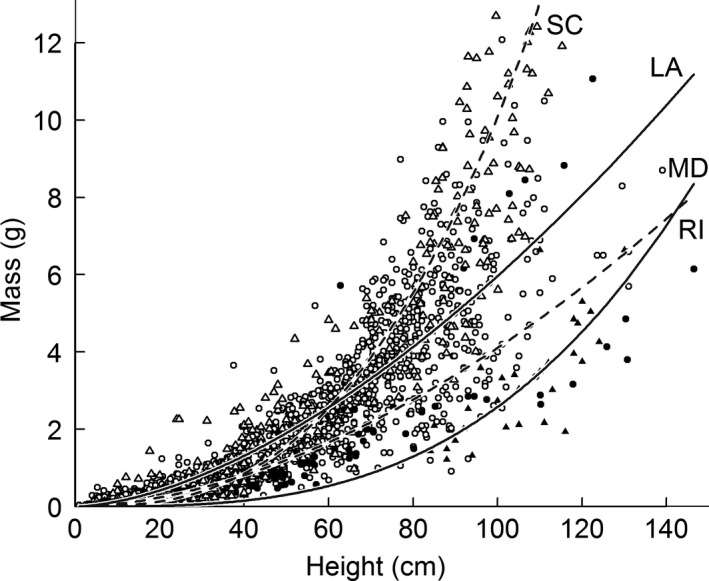
Summertime *S. alterniflora* allometry in Louisiana (open circles, solid line; present study, *n* = 1269), South Carolina (open triangles, dashed line; Morris & Haskin, 1990; *n* = 895), Maryland (filled triangles, solid line; Lu et al., 2016, *n* = 30), and Rhode Island (filled circles, dashed line; T. Hill unpublished and C. Wigand unpublished, *n* = 205). Lines of best fit determined by state‐specific nonlinear regressions based on significant differences in residuals from a pooled model

In addition to the fine‐scale variation described above, large‐scale latitudinal variations in allometry are also present. A continental‐scale allometry model based on growing‐season data had significantly different residuals between states (Figure 4). Latitudinal differences in allometry were sufficient to produce mass estimates that diverge by a factor of two or more. Stems of a given height tended to weigh more at marshes nearer the equator (open vs. closed symbols in Figure 4). The trade‐off between mass at unit height (allometry coefficients) and mass accumulation rates (allometry exponents) described above (Figure 3) also appears in allometry parameters collected from published literature.

### 3.2 Trends in *Spartina alterniflora* biomass and stem density

Monthly sampling resolution in the LUMCON region allows a higher‐resolution presentation of biomass trends, and therefore, the present section focuses primarily on the three LUMCON marshes although patterns were similar across regions.

**Figure 5:**
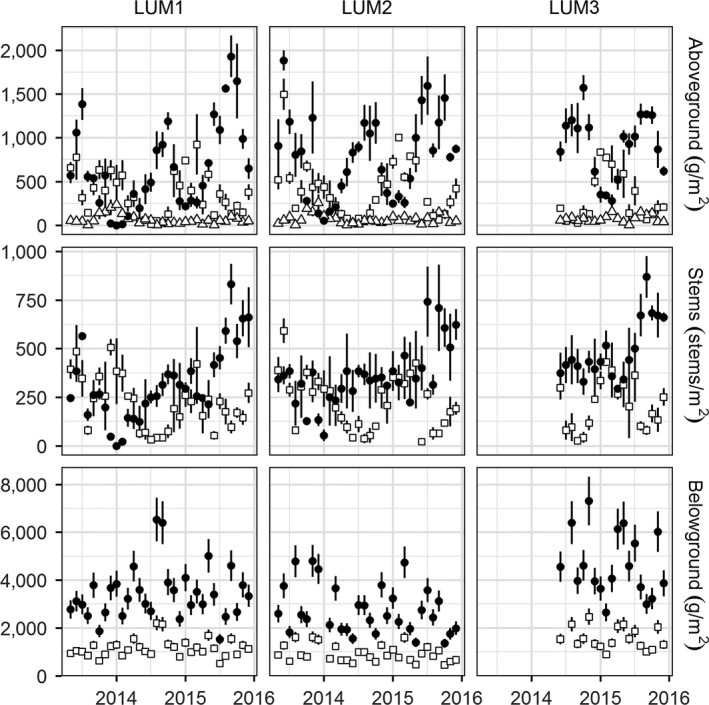
Monthly aboveground biomass (top row), stem density (middle row), and belowground biomass (bottom row) at the three LUMCON marshes. Live, dead, and litter biomass are represented as closed circles, open squares, and open triangles, respectively (mean ± *SE*). Belowground live and dead biomass were separated beginning in May 2015; prior estimates use the relationship established between live/dead and total biomass (Fig. S1)

Live aboveground biomass increased during the growing season although the timing of peak live biomass occurred as early as July and as late as October (LUMCON; Figure 5) or November (Lake Barre; Fig. S3). Standing dead biomass peaked during the dormant season, as did surface litter, which on average was 65 g/m^2^, approximately 5% of total aboveground biomass but during winter months, the proportional contribution rose to 30%.

Abundance of belowground biomass varied substantially, occasionally two‐fold between sampling events. However, the proportion of live biomass was consistent over the time period when live and dead biomass were separated (slope = 0.75, *r* ^2* *^= .71; Fig. S1). Applying this ratio to the earlier data suggests a fairly homogeneous belowground standing crop in the LUMCON region, without a strong seasonal or annual pattern (Figure 5). At Lake Barre, belowground standing crop was highest in early 2015 (Fig. S3), driving the elevated max–min NBPP in that year and leading to a large departure between max–min and Smalley NBPP. At Bay La Fleur, belowground biomass and NBPP both peaked in 2014.

At LUMCON marshes, the winter of 2014–2015 was characterized by incomplete senescence. This left ~250 g/m^2^ of live biomass remaining aboveground and relatively high live‐stem densities (~250 stems/m^2^). During the subsequent 2015 growing season, stem densities were nearly twice as high as during previous years, and the increase in stem densities was most dramatic for small stems (Fig. S5). After correcting for seasonal trends using time series decomposition and a 12‐month loess window, secular (nonseasonal, nonrandom) trends explained 55% of the variance in stem densities for stems less than 25 cm tall, and 45% of the variance in 25‐ to 50‐cm stems. This compares with <3% of the variation in stems taller than 50 cm. Although it is not possible to isolate the contribution of a single warm winter, our data indicate a sustained increase in the density of small stems.

### 3.3 Net aboveground primary production

**Figure 6:**
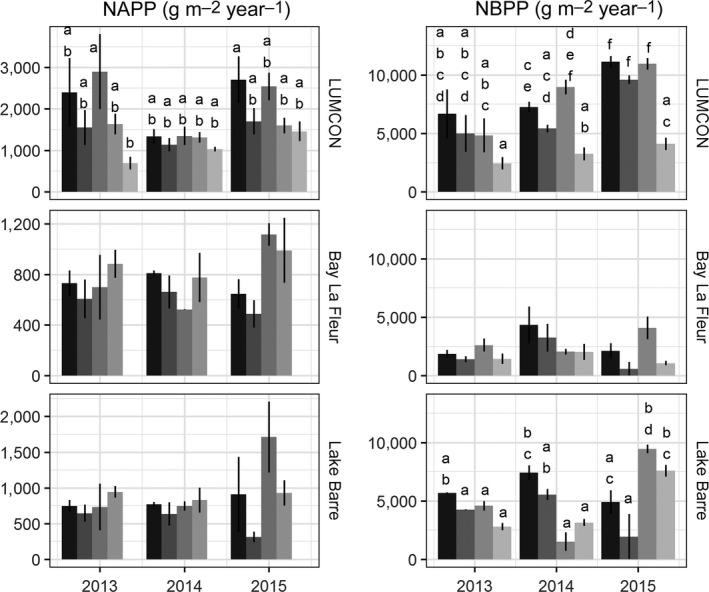
Left side: Five NAPP estimates for the three study regions (mean ± *SE* of 2–3 marshes). Bars from darker to lighter colors: Smalley, Milner‐Hughes, Valiela et al., peak biomass and EOSL. Note different *y*‐axis scales between panels. EOSL is shown only for LUMCON region. Right side: Net belowground primary productivity estimates for each year in the three study regions. Bars from darker to lighter colors: Smalley, Milner‐Hughes, Valiela et al., and max–min. Different letters within each panel indicate significant differences in estimates within a region (type‐III ANOVA with Tukey HSD post hoc test). Absence of letters in a panel indicates no significant differences

For each of the 3 years of the study, NAPP was calculated five ways for each marsh. Although drawing on the same biomass data, estimation methods could vary in magnitude by as much as fourfold (Figure 6). However, primarily when the magnitude of NAPP was low, NAPP estimates within a region were not significantly different across years. Departures between methods were greatest when NAPP was elevated.

Region, year, and calculation method were all significant sources of variation in production NAPP estimates (*F*_2,79 _= 43.5, 7.1, and *F*_4,79 _= 6.6). Smalley NAPP ranged from 390 g m^−2^ year^−1^ at TB3 (Lake Barre) in 2015 to 3,300 at LUM1 in 2015 (Table S6). Productivity was significantly higher in the LUMCON region (2,112 ± 340 g m^−2^ year^−1^; averaging Smalley estimates) than in Bay La Fleur and Lake Barre (729 ± 50 and 809 ± 141), and temporally (averaging across marshes), NAPP was significantly lower in 2014 than in 2015 (Tukey HSD post hoc tests; *p *<* *.05).

In aggregate, NAPP calculation methods largely agreed. The Smalley method was significantly higher than EOSL, and the Valiela method was higher than both EOSL and Milner‐Hughes. Within a region, the only significant differences in NAPP estimates observed were between EOSL and the Smalley and Valiela methods (Figure 6).

Peak and EOSL may overestimate NAPP by assuming complete senescence, or underestimate NAPP by ignoring mortality. Our peak biomass estimates were correlated with Smalley NAPP (Fig. S5; *r* ^2* *^= .72, *p *<* *.001), although this was not the case with the more commonly used NAPP estimate, EOSL (*r* ^2* *^= .14, *p *=* *.35). While this suggests that peak biomass is a reasonable proxy for NAPP, the repeated sampling required to verify that peak biomass was captured would approach that needed for more accurate increment‐based NAPP calculations. Higher‐resolution NAPP estimates based on Milner‐Hughes and Valiela were well‐correlated with Smalley NAPP (*r* ^2 ^= .85 and .84, respectively), although the Valiela method yielded estimates closest in magnitude to Smalley NAPP (slope = 0.90 ± 0.09 vs. 1.52 ± 0.15 for M‐H).

### 3.4 Belowground and total primary production

Belowground production was calculated using four methods: Smalley, Milner‐Hughes, Valiela, and max–min (Figure 6). Analysis of variance indicated significant variation between regions (*F*_2,72_ = 33.0), calculation method (*F*_3,72_ = 8.8), and years (*F*_2,72_ = 7.5). A Tukey post hoc test showed that across years, NBPP was highest at LUMCON, followed by Lake Barre and Bay La Fleur. The only significant differences between calculation methods were that the max–min method was lower than Smalley and Valiela estimates. Averaged across regions and estimation methods, NBPP estimates were significantly higher in 2015 than in 2013 and 2014.

As with NAPP, estimation methods diverged more when NBPP was elevated. At the two higher‐productivity regions, LUMCON and Lake Barre, there were within‐region significant differences between NBPP estimates (Figure 6). The most consistent difference at these marshes was that max–min NBPP was lower than the other methods, particularly Smalley NBPP.

Belowground productivity as calculated by the Smalley method was typically 2.5 times higher than when calculated by the max–min method. Despite the substantial differences in complexity of the two approaches, they were strongly correlated (Fig. S6; *r* ^2^ = .78, *p *<* *.001), with the exception of two outliers (following Lu et al., 2016).

Smalley productivity may be more sensitive to spatial heterogeneity in that it ascribes importance to all variation between sampling events. Max–min estimates are less variable than Smalley productivity, although they rely on fewer data points. Our estimates of total net primary production use what we consider the best estimates of production aboveground (Smalley) and belowground (max–min).

Averaged across all years, max–min belowground productivity was highest at Lake Barre (4,510 ± 990 g m^−2 ^year^−1^) and LUMCON (3,370 ± 367) relative to Bay La Fleur (1,512 ± 280). These values are 5.6, 1.6, and 2.1 times as high as mean NAPP (Smalley) in the respective regions. The below:aboveground ratio at Lake Barre was sufficiently high to yield total primary production (max–min NBPP + Smalley NAPP; Table S6) rates comparable to the LUMCON region, approximately 5,400 g m^−2 ^year^−1^. Combined production in Bay La Fleur was less than half as high as the other regions, ~2,200 g m^−2 ^year^−1^.

### 3.5 Primary production drivers

Soil parameters (bulk density, water content, C, N, and P) were relatively constant through space and time (Table 1), with none varying significantly among regions or years. Growing‐season averages for soil organic C and N varied from 7% to 17% C and 0.6% to 1.7% N. Soil P varied from 510 to 861 ppm.

**Table 1:** Soil and adjacent channel or bay water parameters, averaged from April–October for each region and each year

Region	Year	Soil properties					Channel/Bay water properties (nutrient concentration units: μmol/L)				
		Bulk density (g/cm^3^)	Moisture (%)	Organic C (%)	Total N (%)	Total P (ppm)	Salinity	NO_3_^‐^ + NO_2_^‐^	NH_4_^+^	PO_4_^3‐^	SiO_2_
LUMCON	2013	0.38 (0.02)	75.4 (0.4)	7.2 (1.2)	0.6 (0.0)	510 (5)	9.3 (0.8)	8.5 (0.6)	3.9 (0.4)	1.5 (0.1)	39 (1.3)
LUMCON	2014	0.32 (0.01)	77.0 (0.3)	9.3 (0.8)	0.6 (0.0)	583 (24)	8.5 (0.5)	0.6 (0.1)	3.2 (0.2)	1.5 (0.1)	39.6 (5.7)
LUMCON	2015	0.35 (0.01)	76.0 (0.6)	8.9 (0.4)	1.7 (0.6)	527 (16)	8.5 (0.1)	3.3 (0.4)	10.7 (2.1)	1.3 (0.1)	52.6 (0.7)
Lake Barre	2013	0.30 (0.01)	80.2 (0.8)	12.7 (1.8)	0.9 (0.1)	539 (8)	12.5 (1.0)	1.4 (0.3)	2.2 (0.2)	1.3 (0.1)	33.8 (2.2)
Lake Barre	2014	0.25 (0.03)	81.2 (2.6)	13.7 (3.6)	0.8 (0.1)	589 (25)	14.7 (0.1)	0.6 (0.1)	4.7 (1.5)	0.8 (0.0)	37.1 (1.4)
Lake Barre	2015	0.28 (0.03)	80.1 (1.4)	12.8 (2.2)	0.7 (0.1)	575 (30)	15.0 (0.1)	0.5 (0.0)	0.9 (0.3)	1.0 (0.3)	42.9 (2.1)
Bay La Fleur	2013	0.34 (0.15)	76.6 (9.5)	16.8 (9.5)	1.1 (0.5)	842 (410)	12.0 (2.5)	1.5 (0.1)	0.9 (0.1)	0.7 (0.0)	36.2 (2.4)
Bay La Fleur	2014	0.35 (0.13)	74.8 (7.9)	13.4 (6.7)	0.9 (0.4)	861 (360)	16.1 (0.1)	0.7 (0.4)	1.4 (0.1)	0.6 (0.1)	32.8 (0.6)
Bay La Fleur	2015	0.39 (0.19)	72.7 (7.7)	13.4 (5.8)	0.8 (0.3)	714 (282)	16.8 (0.3)	0.4 (0.1)	0.6 (0.0)	0.3 (0.1)	25.1 (2.5)

Means (*SE* in parentheses) of *n* = 2 marshes, except *n* = 3 for LUMCON in 2014 and 2015.

Water quality parameters were more variable. The salinity of channel or bay water adjacent to the study marshes varied from 9 to 17 psu and varied significantly by region (*F*_2,11_ = 54.9, *p *<* *.001) and year (*F*_2,11_ = 6.0, *p *<* *.05), being significantly lower in the LUMCON region during 2014 and 2015 (Tukey HSD post hoc test). Mean growing‐season NO_3_^‐^ and NH_4_^+^ concentrations were consistently less than 10 μmol/L N, and NO_3_^‐^ had strong regional (*F*_2,11_ = 106.8, *p *<* *.001) and temporal differences (*F*_2,11_ = 74.7, *p *<* *.001), tending to increase over time, with significantly higher concentrations observed at LUMCON. The LUMCON region also had significantly higher NH_4_^+^ concentrations than elsewhere (*F*_2,11_ = 15.1, *p *<* *.001), with no differences over time. Dissolved PO_4_^3‐^ ranged from 0.3 to 1.5 μmol/L P and, as with other dissolved nutrients, was significantly higher at LUMCON (*F*_2,11_ = 55.5, *p *<* *.001) and higher in 2015 than in earlier years (*F*_2,11_ = 11.6, *p *<* *.01). Dissolved Si varied by a factor of two (25–53 μmol/L) and was higher in Lake Barre than in the LUMCON region.

A two‐axis PCA explained a combined 58.2% of the variation in our explanatory variables. The primary PCA dimension accounted for 34.6% of variation in the data and was negatively correlated with soil C and P and salinity in adjacent channels or bays. Dimension 1 was positively correlated with all dissolved nutrient concentrations (Table S7). The PCA secondary dimension was positively correlated with all of the soil parameters (moisture, C, N, and P content).

**Figure 7:**
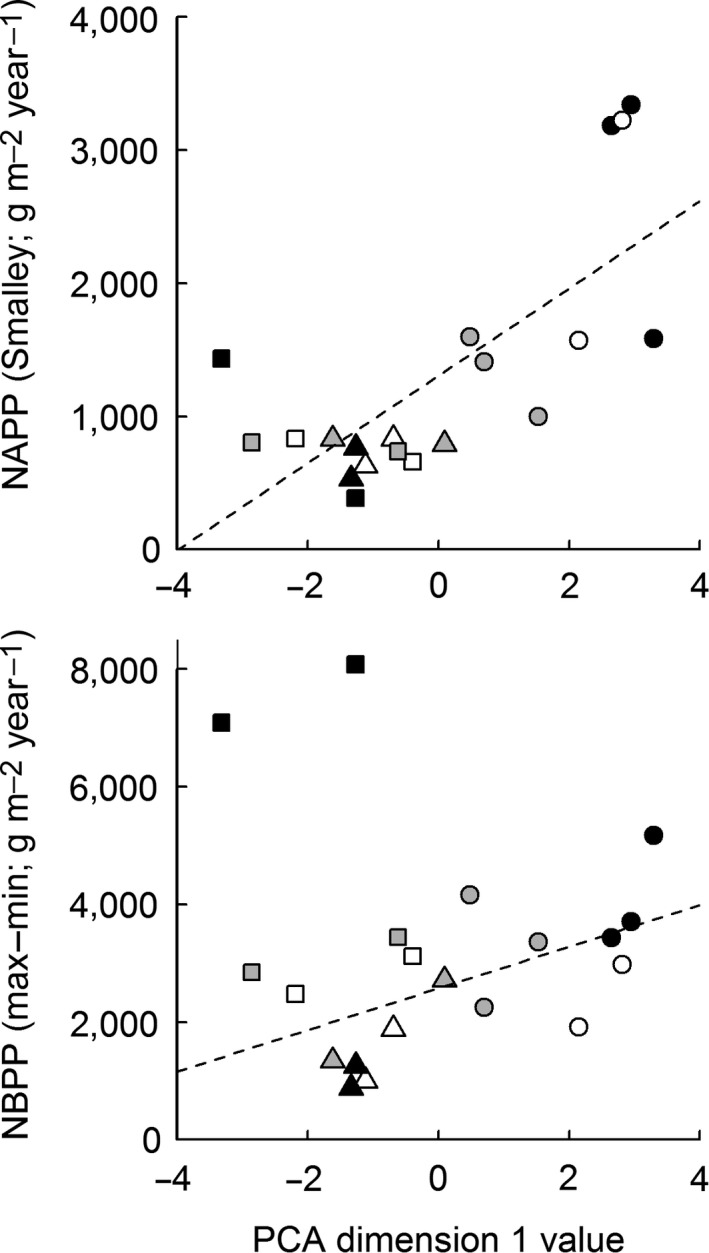
Relationship between productivity (top: Smalley NAPP; bottom: max–min NBPP, excluding two outliers) and principal component analysis dimension 1, characterized primarily by dissolved nutrients and salinity in adjacent bay water (Table S7). Each point represents data from a single marsh in a single year; circles are LUMCON data, triangles are Bay La Fleur, and squares are Lake Barre. Symbol color (white, gray, black) reflects the year (2013–2015, respectively). Lines of best fit: NAPP = 328*x *+ 1306, *r* ^2 ^= .49, *p *<* *.001; NBPP = 355*x *+ 2572, *r* ^2 ^= .30, *p *=* *.01

Only the primary PCA dimension was correlated with primary production, and it explained 50% of the variation in NAPP and 34% of the variation in NBPP (Figure 7). Because values were standardized, variable loadings (Table S7) provide an indication of the relative impact of environmental variables on production. By this measure, PCA dimension 1 had strong negative relationships (loading < −0.30) with creek water salinity and soil C, and strong positive relationships (loading >0.30) with dissolved nutrients. Positive relationships between NAPP/NBPP and PCA dimension 1 indicate that both production measures have positive associations with elevated water column nutrient concentrations, reduced salinities, and low soil C.

## 4 DISCUSSION

### 4.1 Allometry

Our results suggest that mass–height allometry varies on a range of spatial scales. Combined with literature data, we identify a strong latitudinal gradient in allometry, wherein plants from lower latitudes weigh more for a given height. The studies included in this continent‐scale assessment vary not just in latitude and climate, but also in salinity, tidal regimes, and other conditions. The drivers of this variation deserve further investigation, but as a methodological best practice, it appears prudent to develop allometry equations locally.

Even at a local level, the relationship between *S. alterniflora* mass and height varies seasonally and spatially in coastal Louisiana. Stems of a given height weighed more in summer and fall, but the magnitude of the shift varied spatially. Morris and Haskin (1990) report similar seasonality in South Carolina, with stems weighing more in June than in other months and also describe spatial differences in allometry consistent with the variation we observed between regions. Other brief mentions of spatial differences in mass–height allometry also appear in the literature (Hatcher & Mann, 1975; Thursby, Chintala, Stetson, Wigand, & Champlin, 2002). Stem recruitment in coastal Louisiana is somewhat continuous throughout the year (Fig. S4), but stems >50 cm are more abundant in the summer and fall, a possible influence on the detection of seasonality. Tracking of individual stem characteristics, such as stem diameters and leaf:stem mass ratios, as well as population characteristics such as longevity and turnover, could provide a more mechanistic understanding of shifting allometry and its implications for salt marsh carbon dynamics.

Given strong latitudinal and seasonal variation in allometry, nondestructive mass estimates can be greatly improved using locale‐specific allometry built at time intervals aligned with the biomass estimation regime. Allometry equations should also be continuously validated by destructive harvest while mechanisms (salinity, nutrients, flooding regimes) driving allometry variation are explored.

Although allometry may vary through time and space, the shifts are unified by an apparent relationship between allometry coefficients and exponents (Figure 3). This relationship, which appears in allometry equations reported in the literature but has not been previously discussed, suggests a trade‐off between emergent mass of new stems and proportional growth rates. This trade‐off could be an effect of shifting demographics, with periods of high recruitment yielding higher allometry coefficients (mass at unit height). The relationship between allometry coefficients and exponents may also indicate plasticity in plant growth strategies in response to seasonality in environmental conditions. The precise ecological drivers of this plasticity deserve further study, but the strong predictive relationship between growth rates and stem mass at unit height could substantially simplify the development and verification of allometric relationships.

### 4.2 Aboveground primary production and environmental drivers

Results from estimating NAPP using five methods, including snapshot (peak live, EOSL) and increment‐based measures (Milner‐Hughes, Smalley, Valiela), showed reasonable correlations between all estimates except EOSL. Areas of agreement and disagreement between NAPP estimates provide insight into the processes affecting primary production, by virtue of the different accounting approaches used. Peak live biomass, although correlated with more intensive measures, has two associated caveats that limit its applicability as an NAPP estimate. First, variation in the timing of peak biomass (July–November in this study) could cause uncertainty as to whether peak biomass was captured by a September sampling event. Second, the assumptions of a zero‐biomass starting point and a single maximum are increasingly tenuous as warm winters complicate recruitment and growth dynamics and reduce full senescence, as observed in our 2014–2015 data and elsewhere (Day et al., 2013; Hopkinson, Gosselink, & Parrando, 1978).

Snapshot biomass sampling is often used to estimate productivity (Kirwan, Christian, Blum, & Brinson, 2012; Visser et al., 2006), with the unreliable assumption that the two measures are strongly correlated. When studies rely on different sampling regimes and reach disparate conclusions (e.g., Morris et al., 2002 vs. Kirwan et al., 2012), it can be difficult to determine how much of the difference in results is attributable to sampling regimes versus actual ecological processes.

The increment‐based estimates of NAPP may also depart from true production by insufficiently capturing turnover and conflating spatial heterogeneity with biomass changes (Morris & Haskin, 1990). The Milner‐Hughes method ignores simultaneous growth and mortality (Long & Mason, 1983; Turner, 1976). The Smalley and Valiela methods do incorporate mortality by considering changes in dead biomass, but only insofar as‐dead material remains on‐site. These methods do not account for physical biomass removal through, for example, herbivory or tidal export (Long & Mason, 1983).

Agreement between peak live biomass and Milner‐Hughes estimates indicates whether there was a single peak in biomass. Biomass oscillations resulting in relatively higher Milner‐Hughes estimates could be attributed to emergence of new stem cohorts, regrowth after physical disturbance, or simple spatial heterogeneity (Morris & Haskin, 1990). In some cases, peak biomass was greater than Milner‐Hughes estimates (observed at Bay La Fleur and Lake Barre), likely related to assumptions about senescence. Peak and EOSL methods assume complete senescence, whereas Milner‐Hughes does not. If complete senescence is not observed, Milner‐Hughes NAPP will be lower than peak biomass.

Because the Milner‐Hughes approach only considers live biomass increments, differences with Smalley estimates reflect the consideration of dead biomass. The difference between the two methods is the sum of positive dead biomass increments, when dead biomass increments are in excess of declining live biomass accumulation.

Where NAPP estimated by the Smalley and Valiela methods agree, as was largely the case in our data, systems are likely to be in steady state between production and mortality. Divergence between the two methods can reflect a production–mortality imbalance, import or export of biomass between sampling intervals, or artifacts of small‐scale spatial heterogeneity. Instead of creating uncertainty in NAPP, we argue that diverse increment‐based NAPP estimates provide complementary information about biomass dynamics. We consider the Smalley method to provide our most robust estimate of NAPP because it is an increment‐based measure that considers changes in both live and dead material, a preference strengthened by the agreement between the Smalley and Valiela methods.

The extensive literature data on NAPP in Louisiana are dominated by EOSL estimates (e.g., Day et al., 2009; Visser et al., 2006) and often sample in diverse communities (Roberts et al., 2015). Compared with studies that collected high‐resolution data from *S. alterniflora* monocultures, our NAPP estimates for the LUMCON region are substantially higher (Table S6), whereas estimates for Lake Barre and Bay La Fleur were similar to literature values.

Our analysis of environmental drivers suggests that nutrients enhance and salinity inhibits *S. alterniflora* NAPP, consistent with results of manipulative experiments (Morris, Sundberg, & Hopkinson, 2013; Smart & Barko, 1980). The negative effect of salinity on NAPP suggested by our PCA contrasts with Snedden et al. (2015), who found a positive relationship, although their salinity range was lower and narrower (4–8 psu) than ours (7–17 psu). Distinguishing between salinity and nutrient effects is not possible in our data because nutrients were associated with freshwater. However, nutrient and salinity effects are likely to be interactive; reduced salinity allows *S. alterniflora* to more readily access dissolved N and increase growth rates, while moderate increases in dissolved N can ameliorate salinity stress (MacTavish & Cohen, 2017).

Larger‐scale productivity drivers including latitude (integrating temperature and insolation; Kirwan et al., 2009; Turner, 1976) and mean sea level (Morris et al., 2013; Snedden et al., 2015) have also been identified. The effects of these landscape‐scale drivers are largely muted in our data; mean growing‐season sea level and water temperatures were constrained to narrow ranges (2.7–6.8 cm below MSL, and 27.2–28.1°C) during this three‐year study (NOAA station 8761724). With sea level and temperature remaining relatively constant, our results show that nutrients and salinity dominate as drivers of NAPP and can induce dramatic differences in aboveground production.

### 4.3 Belowground production and environmental drivers

Belowground production estimates in the present study were comparable to literature values from *S. alterniflora* marshes along the Gulf Coast and the southeastern USA (Table S6). Although studies rarely report multiple NBPP estimates for the same data, max–min estimates appear generally lower than Smalley estimates, consistent with our data. This is especially evident in studies applying both methods; for example, Darby and Turner (2008b) report Smalley NBPP nearly an order of magnitude higher than max–min.

This disparity can be imparted by repeated oscillations of belowground standing crop within a narrow range, which would accumulate gains to Smalley NBPP while minimally affecting max–min NBPP. Variation in our Smalley NBPP estimates is derived in part from such oscillations rather than strong seasonal patterns, suggesting that spatial heterogeneity in belowground biomass may be high. Smalley NBPP may be artificially inflated by this variation because the method imparts significance to each positive biomass increment. For this reason, we feel that max–min NBPP provides a more meaningful estimate, although it relies on just two values.

As observed with NAPP, NBPP was positively correlated with dissolved nutrients and negatively correlated with salinity of adjacent water. These results fit within a conflicted experimental literature. Fertilization experiments in *S. alterniflora* marshes have reported nitrogen‐associated declines in live belowground biomass (Deegan et al., 2012; *p *=* *.08; Hines, Megonigal, & Denno, 2006; *p *=* *.06), as well as positive or neutral nitrogen effects (Anisfeld & Hill, 2012; Darby & Turner, 2008a; Davey et al., 2011; Valiela, Teal, & Persson, 1976). Darby and Turner (2008a) report declines in live biomass stocks in response to P and Fe amendments, but this contrasts with other P fertilization work (Anisfeld & Hill, 2012; Davey et al., 2011). Adding further complexity, the distinction between standing stocks and production is often neglected in plot‐scale experiments that have limited space available for the required sequential coring.

In addition to the distinction between production‐ and snapshot‐based measures of biomass stocks, the magnitude of nutrient loading may play a role in contextualizing our results with fertilization studies that report negative impacts. For example, dissolved inorganic nitrogen concentrations in our creeks (interquartile range of 2.9–10.7 μmol/L DIN) are an order of magnitude lower than those used in the Plum Island Ecosystem (PIE) creek fertilization experiment (70–100 μmol/L NO_3_‐N in Deegan et al., 2012). Johnson, Warren, Deegan, and Mozdzer (2016) estimate that 171 g N m^−2 ^year^−1^ is delivered to the low marsh by the PIE creek fertilization treatment, although even this rate is lower than N applications in many plot‐scale experiments, such as the 370 g N m^−2 ^year^−1^ applied by Darby and Turner (2008a), where nitrogen alone had no effect on belowground production. Our results indicate that, at current ambient concentrations observed in coastal Louisiana, natural variation in nutrients is more likely to enhance than inhibit belowground production.

Observational studies along combined nutrient–freshwater gradients support a positive association between NBPP and reduced salinity. Large‐scale freshwater diversions such as the Caernarvon diversion of Mississippi River water into Breton Sound (Day et al., 2013; Snedden et al., 2015) suggest that belowground biomass stocks are affected primarily by flooding stress rather than by nutrient/salinity effects of freshwater inputs. However, rapid nutrient uptake near river diversions reduces nutrient concentrations downstream (Day et al., 2013), leading these studies to primarily document effects of salinity and flooding regimes.

Unlike large‐scale diversions, the salinity and nutrient gradients in the present study are more strongly coupled and are driven by natural variation in precipitation and watershed runoff. The Intergovernmental Panel on Climate Change estimates that growing‐season precipitation in coastal Louisiana may increase up to 20% during the twenty‐first century (75% percentile, RCP4.5 scenario; IPCC, 2013). The present study suggests that any increase in precipitation and runoff could increase salt marsh carbon fixation above‐ and belowground. If increased carbon fixation is not offset by more rapid decomposition, the net result would be enhanced peat development, which could increase resilience to submergence and provide a regulating effect on climate change as atmospheric CO_2_‐C is stored in marsh peat.

## DATA ACCESSIBILITY

Data are publicly available through the Gulf of Mexico Research Initiative Information & Data Cooperative (GRIIDC) at https://data.gulfresearchinitiative.org (https://doi.org/10.7266/n72v2d1t, https://doi.org/10.7266/n7z31wkw, https://doi.org/10.7266/n7tm7858, https://doi.org/10.7266/n7k35rq4).

## CONFLICT OF INTEREST

None declared.

## AUTHORS’ CONTRIBUTIONS

BR conceived the ideas and designed methodology; BR and TH collected the data; TH analyzed the data; TH and BR wrote the manuscript.
