# Supplementary material for: Effects of seasonality and environmental gradients on Spartina alterniflora allometry and primary production
Source: Ecol Evol. 2017 Oct 16;7(22):9676–88. doi: 10.1002/ece3.3494 (PMC5696422; doi:10.1002/ece3.3494)
Supplement: Supplementary file 1 [file ECE3-7-9676-s001.docx]

**Supplementary Information for Hill and Roberts**

1. Results of ANOVAs of residuals from stem allometry models are below. Region categories were LUMCON (LUM), Lake Barre (TB-A), Bay La Fleur (TB-B). Sampling times were classified into four seasons: winter (December – February), spring (March – May), summer (June – August), and fall (September – November).

Table S1. ANOVA table for residuals of pooled live-stem allometry model.

|  | Sum of sq (Type III) | Df | F value | Pr (>F) |  |
| --- | --- | --- | --- | --- | --- |
| (Intercept) | 2.33 | 1 | 3.34 | 0.068 | . |
| **season** | **117.85** | **3** | **56.26** | **<2E-16** | ******* |
| **region** | **7.56** | **2** | **5.41** | **0.0045** | ****** |
| **season:region** | **31.48** | **6** | **7.51** | **5.04E-08** | ******* |
| Residuals | 2544.25 | 3644 |  |  |  |

Table S2. Regression coefficients, exponents, and *r^2^* from allometry models relating stem masses to heights. Parameters are specified for each of three regions. For live stems, parameters are reported for each of four seasons; dead stem allometry did not vary and data were pooled across regions and seasons. All models are statistically significant at P < 0.001.

| Region | Season | Type | coefficient | exponent | *r^2^* |
| --- | --- | --- | --- | --- | --- |
| LUM | Spring | Live | 0.0001 | 2.395 | 0.72 |
| LUM | Summer | Live | 0.0028 | 1.676 | 0.62 |
| LUM | Fall | Live | 0.0022 | 1.694 | 0.75 |
| LUM | Winter | Live | 0.0024 | 1.625 | 0.71 |
| Bay La Fleur | Spring | Live | 0.0001 | 2.399 | 0.63 |
| Bay La Fleur | Summer | Live | 0.0011 | 1.881 | 0.73 |
| Bay La Fleur | Fall | Live | 0.0002 | 2.161 | 0.81 |
| Bay La Fleur | Winter | Live | 0.0029 | 1.547 | 0.69 |
| Lake Barre | Spring | Live | 0.0013 | 1.765 | 0.32 |
| Lake Barre | Summer | Live | 0.0074 | 1.413 | 0.32 |
| Lake Barre | Fall | Live | 0.0010 | 1.904 | 0.74 |
| Lake Barre | Winter | Live | 0.0030 | 1.559 | 0.52 |
| All | All | Dead | 0.0017 | 1.679 | 0.51 |

Table S3. ANOVA comparisons for residuals of pooled dead-stem allometry model.

|  | Sum of sq (Type III) | Df | F value | Pr (>F) |  |
| --- | --- | --- | --- | --- | --- |
| **(Intercept)** | **2.17** | **1** | **4.6392** | **0.03135** | ***** |
| season | 3.64 | 3 | 2.5894 | 0.05132 | . |
| region | 0.24 | 2 | 0.2583 | 0.77241 |  |
| season:region | 1.72 | 6 | 0.6118 | 0.72106 |  |
| Residuals | 1066.73 | 2279 |  |  |  |

1. Estimation of belowground live biomass

Prior to May 2015, live and dead belowground biomass were not separated. Live and dead biomass during that period were estimated using the strong relationship observed between total and live belowground biomass when live biomass was separated (Fig. S1).


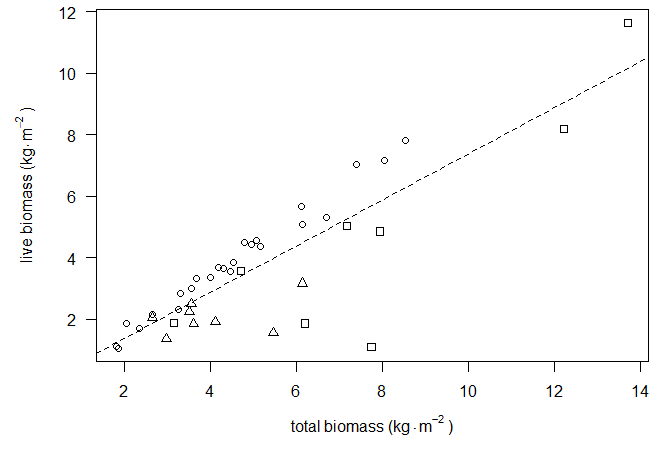


Fig. S1. Relationship between live belowground biomass and total belowground biomass from samples collected from May-Dec 2015. Dashed line is line of best fit (y = 0.75x – 0.11; *r^2^* = 0.71, *P* < 0.001). Each point represents data from an individual marsh in a single month; circles are LUMCON data, triangles are Bay La Fleur, and squares are Lake Barre.

1. Effect of flowering on allometry and detection of seasonality


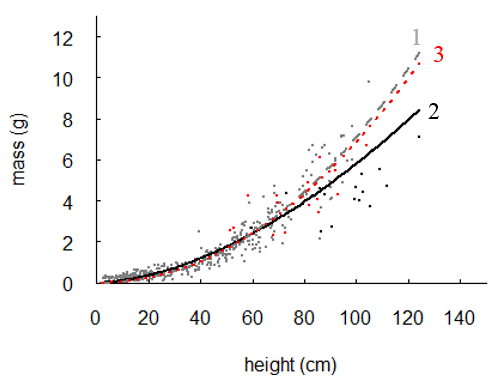


Fig. S2. Effect of flowering on mass-height allometry during fall of 2015 in the LUMCON region. Dataset 1 excludes flowering entirely, showing only non-flowering plants and associated allometry curve as gray points and gray dashed line. Dataset 2 includes n = 20 flowering plants (black points) and allometry of combined data (black line). Dataset 3 corrects flowering stems for the mass and height of inflorescences (red points show corrected values for flowering stems), with allometry shown as a dashed red line.

Table S4. ANOVA results for residuals from a pooled (all regions, all seasons) 2015 allometry model. Table compares analysis under three scenarios: (1) raw data, including flowering stems, (2) correcting flowering stem masses and heights for flower mass/length, and (3) excluding flowering stems entirely. In each case, season is a significant main effect.

|  | Raw data | | | | Correcting flowering stems | | | | Excluding flowering stems | | | |
| --- | --- | --- | --- | --- | --- | --- | --- | --- | --- | --- | --- | --- |
|  | SS | Df | F | P | SS | Df | F | P | SS | Df | F | P |
| **season** | **85.7** | **3** | **56** | **<0.01** | **86.2** | **3** | **66** | **<0.01** | **86.4** | **3** | **71** | **<0.01** |
| region | 0.4 | 1 | 1 | 0.17 | 0 | 1 | 0 | 0.95 | 0 | 1 | 0 | 0.98 |
| season:region | 3.3 | 3 | 2 | 0.10 | 0.5 | 3 | 0 | 0.75 | 1.6 | 3 | 1 | 0.26 |
| Residuals | 731.7 | 1427 |  |  | 621.0 | 1427 |  |  | 553.7 | 1371 |  |  |

Table S5. Results of Tukey post-hoc test on season main effect for 2015 mass-height data. Table compares analysis under three scenarios: (1) raw data, including flowering stems, (2) correcting flowering stem masses and heights for flower mass/length, and (3) excluding flowering stems entirely. The weakest difference (fall-winter) is the one most affected by flowering, and correcting for flower heights and masses leads to an insignificant difference in these seasons. Still, the magnitude of differences and their significance levels are comparable in all three scenarios, indicating that seasonal differences persist irrespective of flowering.

|  | Raw data | | | Correcting flowering stems | | | Excluding flowering stems | | |
| --- | --- | --- | --- | --- | --- | --- | --- | --- | --- |
| Comparison (seasons) | Diff. | *P* (adjusted) | | Diff. | *P* (adjusted) | | Diff. | *P* (adjusted) | |
| **sprg-wint** | **-0.39** | **< 0.001** | ******* | **-0.64** | **< 0.001** | ******* | **-0.62** | **< 0.001** | ******* |
| **sumr-wint** | **0.60** | **< 0.001** | ******* | **0.45** | **< 0.001** | ******* | **0.46** | **< 0.001** | ******* |
| **fall-wint** | **0.17** | **0.010** | ***** | 0.13 | 0.056 | . | **0.13** | **0.043** | ***** |
| **sumr-sprg** | **0.99** | **< 0.001** | ******* | **1.09** | **< 0.001** | ******* | **1.08** | **< 0.001** | ******* |
| **fall-sprg** | **0.56** | **< 0.001** | ******* | **0.77** | **< 0.001** | ******* | **0.76** | **< 0.001** | ******* |
| **fall-sumr** | **-0.43** | **< 0.001** | ******* | **-0.32** | **< 0.001** | ******* | **-0.33** | **< 0.001** | ******* |

1. Primary production and stem dynamics


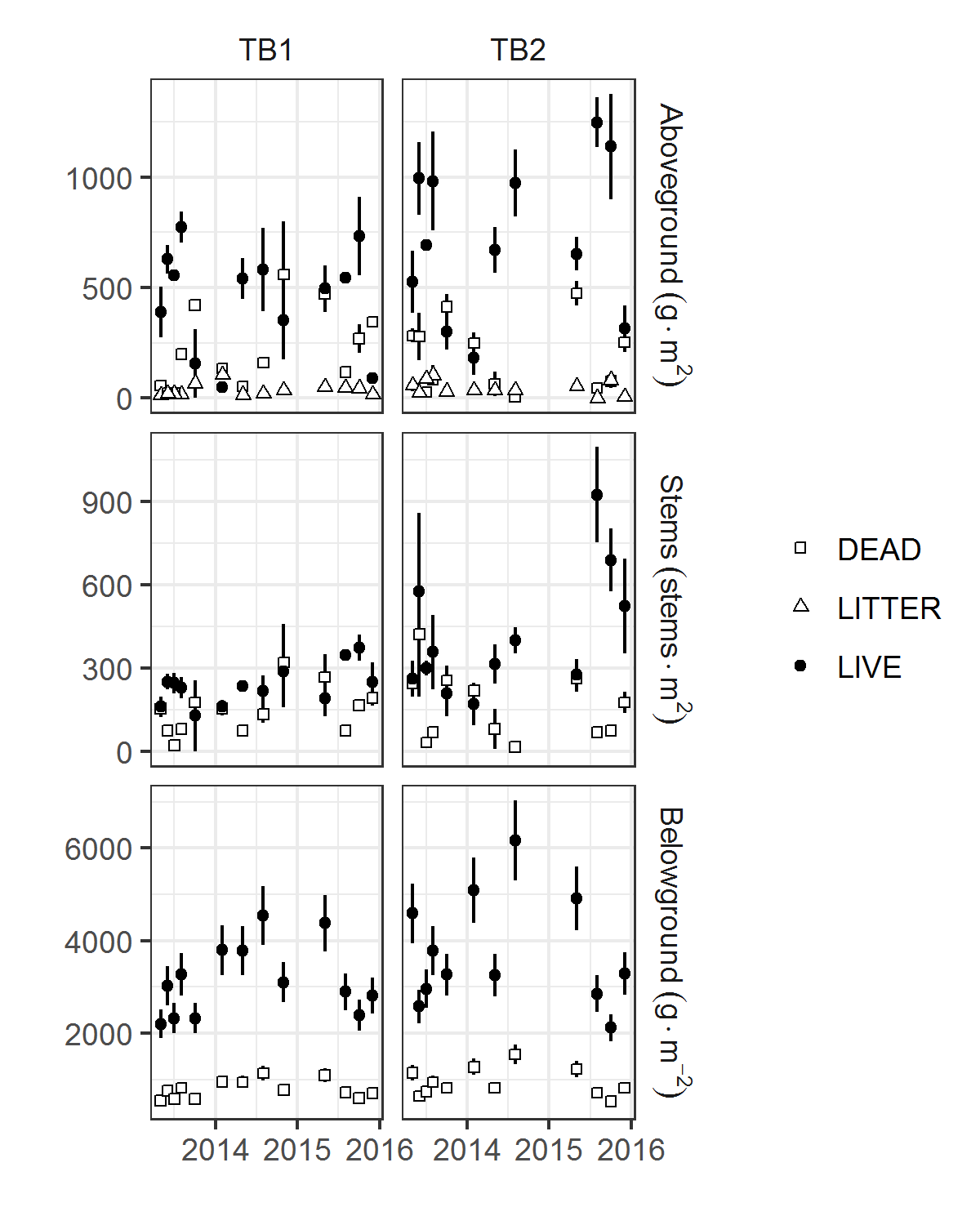

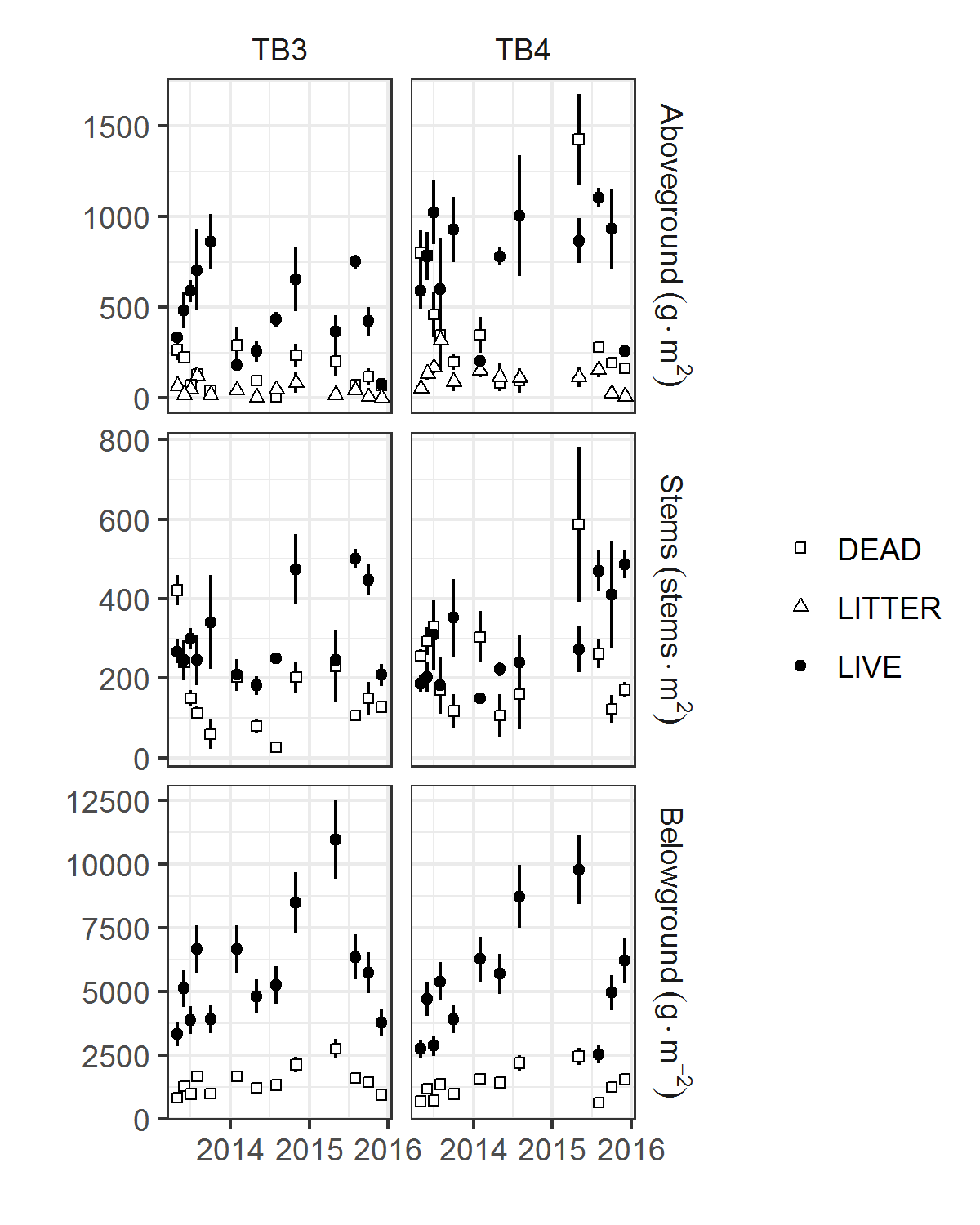


Fig. S3. Aboveground biomass, stem density, and belowground biomass at Bay La Fleur (left side) and Lake Barre (right side) marshes. Points shown are mean ± SE of three plots. Live, dead, and litter biomass are represented as closed circles, open squares, and open triangles, respectively.


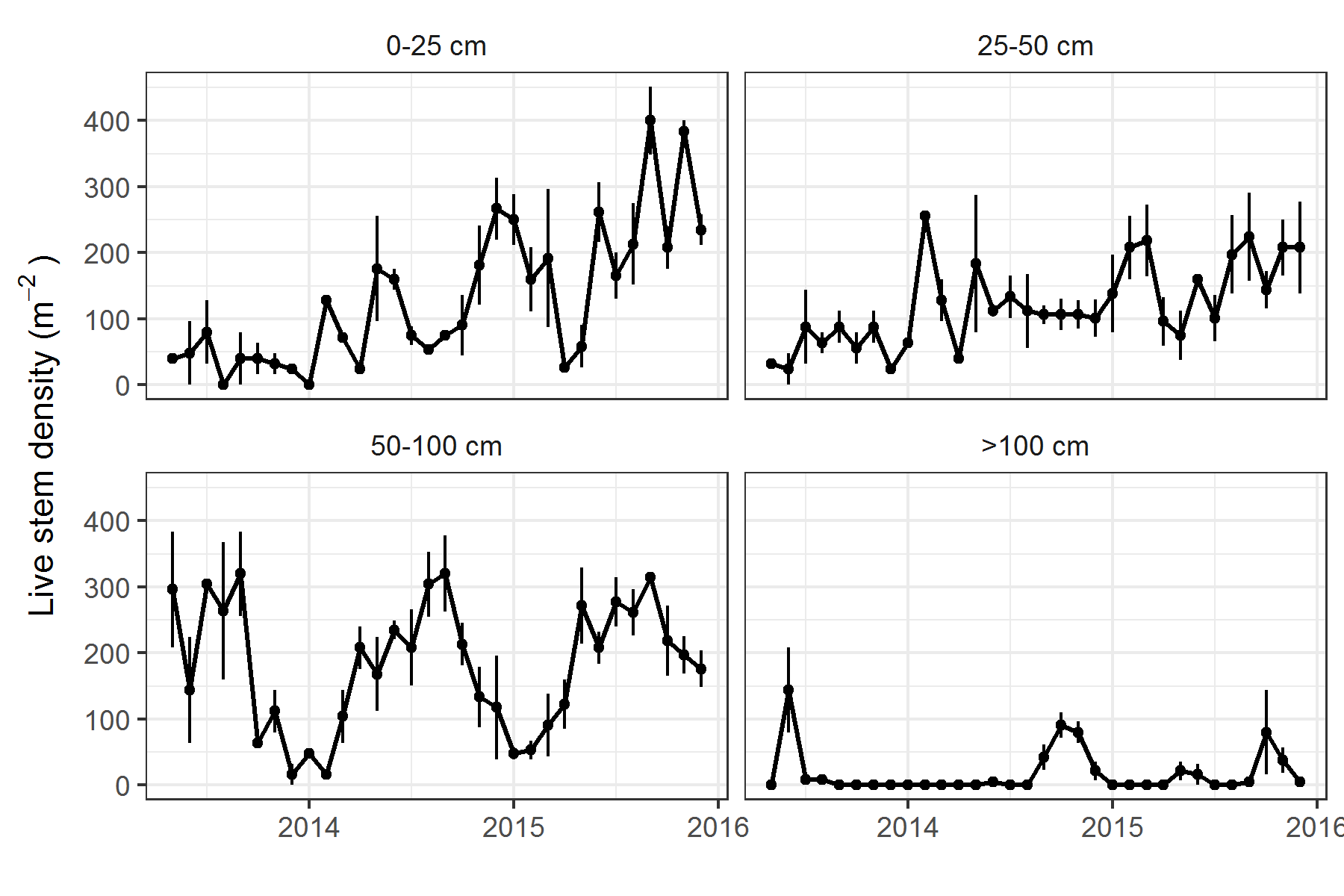


Fig. S4. Time trends in density of live stems at LUMCON. Stems were binned into size classes, shown in panels.

Fig. S5. Relationships between Smalley NAPP and other NAPP estimates. Each point represents a single marsh in a single year. Solid 1:1 line is shown in black. Lines of best fit are shown when significant (*P* < 0.05; all except EOSL). Milner-Hughes regression line slope is 1.52 ± 0.15 (*r^2^* = 0.85), Valiela slope is 0.90 ± 0.09 (*r^2^* = 0.83), and peak biomass slope is 2.01 ± 0.29 (*r^2^* = 0.71).


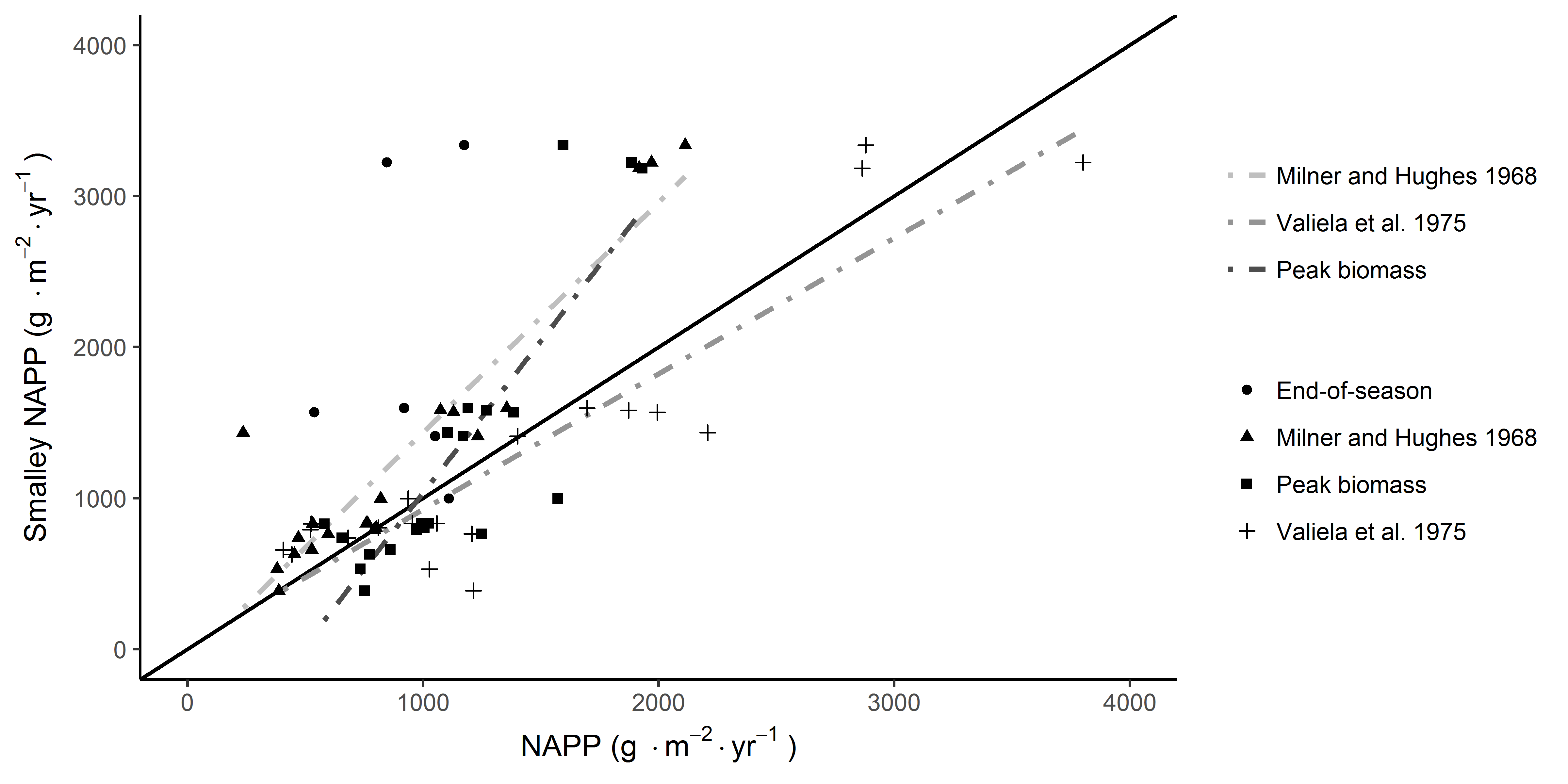


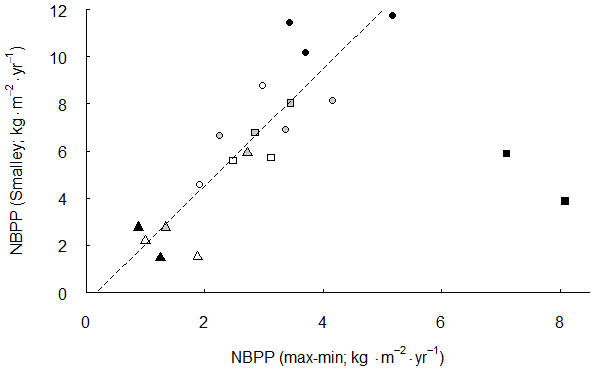


Fig. S6. Relationship between max-min and Smalley belowground production estimates and line of best fit excluding two outliers (y = 2.48x - 0.43, *r^2^* = 0.78). Each point represents data from a single marsh in a single year; circles are LUMCON data, triangles are Bay La Fleur, and squares are Lake Barre. Symbol color (white, gray, black) reflects the year (2013- 2015, respectively). Outliers (excluded from line of best fit) are black squares. Max-min and Smalley estimates are strongly correlated but differ in magnitude.

Table S6. Aboveground and belowground primary production estimates from Louisiana. Total production is sum of aboveground (Smalley) and belowground (max-min) productivities. Data from marshes in the current study are shown in bold. Italics indicate estimates derived from figures using DataThief III. ^1^mean of two salt marshes. ^2^in-growth method used

|  |  |  | NAPP | | | | | NBPP | | Total production |
| --- | --- | --- | --- | --- | --- | --- | --- | --- | --- | --- |
| Location | Time period | Source | Smalley | Milner-Hughes | Valiela | Peak live | EOSL | Smalley | Max-min | Smalley NAPP + max-min BNPP |
| **Cocodrie, LA** | **2013** | **this study (LUM1)** | **1569** | **1129** | **1994** | **1385** | **538** | **4576** | **1916** | **3485** |
| **Cocodrie, LA** | **2014** | **this study (LUM1)** | **1596** | **1354** | **1696** | **1190** | **920** | **8137** | **4163** | **5759** |
| **Cocodrie, LA** | **2015** | **this study (LUM1)** | **3185** | **1916** | **2863** | **1930** | **1930** | **11446** | **3432** | **6617** |
| **Cocodrie, LA** | **2013** | **this study (LUM2)** | **3222** | **1969** | **3800** | **1884** | **847** | **8771** | **2976** | **6198** |
| **Cocodrie, LA** | **2014** | **this study (LUM2)** | **1410** | **1232** | **1401** | **1169** | **1051** | **6666** | **2244** | **3654** |
| **Cocodrie, LA** | **2015** | **this study (LUM2)** | **3338** | **2113** | **2879** | **1594** | **1175** | **10162** | **3705** | **7043** |
| **Cocodrie, LA** | **2014** | **this study (LUM3)** | **997** | **819** | **936** | **1571** | **1109** | **6932** | **3356** | **4353** |
| **Cocodrie, LA** | **2015** | **this study (LUM3)** | **1582** | **1074** | **1873** | **1268** | **1267** | **11755** | **5171** | **6753** |
| **Bay La Fleur, LA** | **2013** | **this study (TB1)** | **628** | **454** | **443** | **772** | **-** | **2204** | **1000** | **1628** |
| **Bay La Fleur, LA** | **2014** | **this study (TB1)** | **830** | **531** | **525** | **581** | **-** | **2770** | **1342** | **2172** |
| **Bay La Fleur, LA** | **2015** | **this study (TB1)** | **531** | **381** | **1027** | **733** | **-** | **2769** | **878** | **1409** |
| **Bay La Fleur, LA** | **2013** | **this study (TB2)** | **832** | **759** | **954** | **994** | **-** | **1519** | **1884** | **2716** |
| **Bay La Fleur, LA** | **2014** | **this study (TB2)** | **792** | **792** | **521** | **972** | **-** | **5915** | **2716** | **3508** |
| **Bay La Fleur, LA** | **2015** | **this study (TB2)** | **764** | **596** | **1206** | **1248** | **-** | **1468** | **1253** | **2017** |
| **Lake Barre, LA** | **2013** | **this study (TB3)** | **658** | **527** | **406** | **862** | **-** | **5729** | **3117** | **3775** |
| **Lake Barre, LA** | **2014** | **this study (TB3)** | **737** | **471** | **681** | **655** | **-** | **8046** | **3445** | **4182** |
| **Lake Barre, LA** | **2015** | **this study (TB3)** | **388** | **388** | **1214** | **753** | **-** | **3891** | **8081** | **8469** |
| **Lake Barre, LA** | **2013** | **this study (TB4)** | **833** | **765** | **1058** | **1025** | **-** | **5607** | **2478** | **3311** |
| **Lake Barre, LA** | **2014** | **this study (TB4)** | **804** | **800** | **809** | **1006** | **-** | **6776** | **2847** | **3651** |
| **Lake Barre, LA** | **2015** | **this study (TB4)** | **1434** | **237** | **2208** | **1105** | **-** | **5906** | **7090** | **8524** |
| Breton Sound, LA | 2006 | Day et al. 2013^1^ | - | *768* | *768* | *1237* | *895* | - | *7507* | - |
| Cocodrie, LA | 2004-2005 | Darby and Turner 2008 | 1281 | 762 | *972* | 877 | 877 | 11676 | 1947 | 3228 |
| Airplane Lake, LA | 1980-1981 | Kaswadji et al. 1990 | 1231 | 831 | *1037* | 831 | *532* | - | - | - |
| Airplane Lake, LA | 1970-1971 | Kirby and Gosselink 1976 | 1410 | 874 | *1158* | 1018 | 1018 | - | - | - |
| Caminada Pass, LA | 1973-1975 | Hopkinson et al. 1978 | *1156* | *477* | *807* | 678 | *593* | - | - | - |
| Black Bay, LA | 1975-1976 | White et al. 1978 | *1198* | *736* | *509* | *1047* | *944* | - | *164* | 1363 |
| Barataria Bay, LA | 1973 | Patrick and Delaune 1976 | - | - | - | 1666 | - | - | - | - |
| Barataria Bay, LA | 1976 | Delaune et al. 1979 | - | - | - | 1679 | - | - | - | - |
| Sapelo Island, GA | 1980 | Schubauer and Hopkinson 1984 | 2840 | *575* | - | 733 | - | 4780 | *-* | - |
| Sapelo Island, GA | 1972-74 | Gallagher and Plumley 1979, Gallagher et al. 1980 | 3700 | - | - | *1371* | *1171* | - | 2100 | 5800 |
| Sabine Pass, LA | 2002 | Edwards and Mills 2005 (NAT2) | - | - | - | *607* | *600* | - | 2332^2^ | - |

1. Primary production drivers: principal components analysis

Table S7. Correlations between explanatory variables and PCA dimensions, and variable loadings along PCA dimension 1. Dimension 1 loadings are shown because a significant relationship existed between dimension 1 and NAPP.

| Parameter | Correlation with dimension 1 | Correlation with dimension 2 | Loading (dimension 1) |
| --- | --- | --- | --- |
| Year | n.s. | n.s. | 0.02 |
| Soil moisture content | n.s. | 0.78 | -0.16 |
| Soil C (%) | -0.68 | 0.68 | -0.35 |
| Soil N (%) | n.s. | 0.79 | 0.10 |
| Soil P (mg gdw^-1^) | -0.57 | 0.64 | -0.29 |
| Bay water temperature | n.s. | n.s. | -0.13 |
| Bay water salinity | -0.79 | n.s. | -0.40 |
| PO_4_-P (µM) | 0.83 | n.s. | 0.43 |
| NO_3_-N (µM) | 0.63 | n.s. | 0.32 |
| NH_4_-N (µM) | 0.76 | n.s. | 0.39 |
| SiO_2_-Si (µM) | 0.73 | n.s. | 0.37 |

Literature cited

Darby, F.A. and Turner, R.E., 2008. Below- and aboveground Spartina alterniflora production in a Louisiana salt marsh. Estuaries and Coasts, 31(1): 223-231.

Day, J., Lane, R., Moerschbaecher, M., DeLaune, R., Mendelssohn, I., Baustian, J. and Twilley, R., 2013. Vegetation and soil dynamics of a Louisiana estuary receiving pulsed Mississippi River water following Hurricane Katrina. Estuaries and Coasts, 36(4): 665-682.

Delaune, R.D., Buresh, R.J. and Patrick, W.H., 1979. Relationship of soil properties to standing crop biomass of Spartina alterniflora in a Louisiana marsh. Estuarine and Coastal Marine Science, 8(5): 477-487.

Edwards, K.R. and Mills, K.P., 2005. Aboveground and belowground productivity of Spartina alterniflora (smooth cordgrass) in natural and created Louisiana salt marshes. Estuaries, 28(2): 252-265.

Gallagher, J.L. and Plumley, F.G., 1979. Underground biomass profiles and productivity in Atlantic coastal marshes. American Journal of Botany, 66(2): 156-161.

Gallagher, J.L., Reimold, R.J., Linthurst, R.A. and Pfeiffer, W.J., 1980. Aerial production, mortality, and mineral accumulation-export dynamics in Spartina alterniflora and Juncus roemerianus plant stands in a Georgia salt marsh. Ecology, 61(2): 303-312.

Hopkinson, C.S., Gosselink, J.G. and Parrando, R.T., 1978. Aboveground production of seven marsh plant species in coastal Louisiana. Ecology, 59(4): 760-769.

Kaswadji, R.F., Gosselink, J.G. and Turner, R.E., 1990. Estimation of primary production using five different methods in a Spartina alterniflora salt marsh. Wetlands Ecology and Management, 1(2): 57-64.

Kirby, C.J. and Gosselink, J.G., 1976. Primary production in a Louisiana Gulf Coast Spartina alterniflora marsh. Ecology, 57(5): 1052-1059.

Patrick, W.H. and Delaune, R.D., 1976. Nitrogen and phosphorus utilization by Spartina alterniflora in a salt marsh in Barataria Bay, Louisiana. Estuarine and Coastal Marine Science, 4(1): 59-64.

Schubauer, J.P. and Hopkinson, C.S., 1984. Aboveground and belowground emergent macrophyte production and turnover in a coastal marsh ecosystem, Georgia. Limnology and Oceanography, 29(5): 1052-1065.

White, D.A., Trapani, J.M., Thien, L.B. and Weiss, T.E., 1978. Productivity and decomposition of dominant salt marsh plants in Louisiana. Ecology, 59(4): 751-759.
